# Implementation Science Perspectives on Implementing Telemedicine Interventions for Hypertension or Diabetes Management: Scoping Review

**DOI:** 10.2196/42134

**Published:** 2023-03-14

**Authors:** Ayisha Khalid, Quanfang Dong, Enkhzaya Chuluunbaatar, Victoria Haldane, Hammad Durrani, Xiaolin Wei

**Affiliations:** 1 Dalla Lana School of Public Health University of Toronto Toronto, ON Canada

**Keywords:** telemedicine, hypertension, diabetes, implementation science, mobile phone

## Abstract

**Background:**

Hypertension and diabetes are becoming increasingly prevalent worldwide. Telemedicine is an accessible and cost-effective means of supporting hypertension and diabetes management, especially as the COVID-19 pandemic has accelerated the adoption of technological solutions for care. However, to date, no review has examined the contextual factors that influence the implementation of telemedicine interventions for hypertension or diabetes worldwide.

**Objective:**

We adopted a comprehensive implementation research perspective to synthesize the barriers to and facilitators of implementing telemedicine interventions for the management of hypertension, diabetes, or both.

**Methods:**

We performed a scoping review involving searches in Ovid MEDLINE, Embase, CINAHL, Cochrane Library, Web of Science, and Google Scholar to identify studies published in English from 2017 to 2022 describing barriers and facilitators related to the implementation of telemedicine interventions for hypertension and diabetes management. The coding and synthesis of barriers and facilitators were guided by the Consolidated Framework for Implementation Research.

**Results:**

Of the 17,687 records identified, 35 (0.2%) studies were included in our scoping review. We found that facilitators of and barriers to implementation were dispersed across the constructs of the Consolidated Framework for Implementation Research. Barriers related to cost, patient needs and resources (eg, lack of consideration of language needs, culture, and rural residency), and personal attributes of patients (eg, demographics and priorities) were the most common. Facilitators related to the design and packaging of the intervention (eg, user-friendliness), patient needs and resources (eg, personalized information that leveraged existing strengths), implementation climate (eg, intervention embedded into existing infrastructure), knowledge of and beliefs about the intervention (eg, convenience of telemedicine), and other personal attributes (eg, technical literacy) were the most common.

**Conclusions:**

Our findings suggest that the successful implementation of telemedicine interventions for hypertension and diabetes requires comprehensive efforts at the planning, execution, engagement, and reflection and evaluation stages of intervention implementation to address challenges at the individual, interpersonal, organizational, and environmental levels.

## Introduction

### Background

Noncommunicable diseases (NCDs), such as cardiovascular disease, diabetes, cancer, and respiratory disease, are a leading cause of death and disability worldwide [1]. A total of 41 million deaths worldwide are attributed to NCDs each year, with 20 million of them attributable to hypertension and diabetes alone [2]. NCDs also contribute to a considerable global burden of disease, with millions living with undiagnosed, untreated, or poorly managed hypertension, diabetes, or both [3,4]. By 2030, the number of people living with hypertension and diabetes is projected to reach 1.6 billion and 643 million, respectively [5,6].

There has been ambitious global momentum to address the growing burden of hypertension and diabetes. Targets set out at the 75th World Health Assembly in 2022 aim to diagnose 80% of people living with diabetes and support 80% of people with diabetes to have good control of their blood pressure [7]. However, reaching these goals is challenging across many dimensions. The diagnosis, management, and treatment of hypertension and diabetes are often lengthy and expensive [8]. Accessing and affording care is often challenging for patients and their families [9,10]. Providing high-quality care also draws on considerable health system resources in both low- and high-resource settings [11,12]. Increasingly, people are also being diagnosed with hypertension and diabetes simultaneously, known as co- or multimorbidity, which further complicates management and treatment [13].

The COVID-19 pandemic has highlighted the need for innovative health services to support the growing number of people with NCDs. The pandemic disrupted prevention activities and care for both hypertension and diabetes, exacerbating the existing burden of disease and unmet treatment needs [14,15]. Optimal care for hypertension and diabetes requires routine contact with health care providers (HCPs) for screening, education, medication review and renewals, management of complications, and mental health support, among other things [16,17]. Prolonged lockdowns, stress, an increase in working from home, and rising food insecurity have increased people’s risk of NCDs and their sequelae through compromised nutrition, limited physical activity, and disrupted access to care [16,18-20]. Thus, there is an urgent need for innovative NCD service delivery and a nuanced understanding of its implementation [14].

One such innovation is telemedicine [21]. Already, telemedicine is being used to effectively manage and treat patients living with hypertension, diabetes, or both across low- and high-resource settings [22-27]. The COVID-19 pandemic has accelerated the adoption of telemedicine for routine health service delivery [28-30]. Given its relatively low-cost implementation and previous successes, telemedicine is a promising, equitable approach for improving access to and ensuring continuity of care in low- and high-resource settings alike [23,31,32].

### Objectives

Existing reviews have identified features that can make telemedicine interventions more effective at providing care to people living with hypertension or diabetes. However, few have offered insight into the challenges of implementing telemedicine interventions [33-35]. To our knowledge, no review has adopted an implementation science lens to review these interventions. Thus, using a comprehensive implementation research perspective, we aimed to synthesize the current available evidence on the barriers to and facilitators of implementing telemedicine interventions for the management of hypertension, diabetes, or both.

## Methods

### Conceptual Framework

Implementation science seeks to understand how health service interventions are applied and taken up in real-world contexts [36]. Theoretical perspectives have been used to better understand how and why implementation succeeds or fails [37,38]. In this review, we used the Consolidated Framework for Implementation Research (CFIR) to organize data extraction and synthesize our findings [39]. The CFIR provides a standardized structure for aggregating findings from multilevel contexts across diverse disciplines [39]. The CFIR is composed of 39 implementation-related constructs divided into 5 major domains: intervention characteristics, outer setting, inner setting, characteristics of the individuals involved, and process of implementation [39].

Our scoping review followed the 5-stage method outlined by Arksey and O’Malley [40]. We also followed the guidelines described in the PRISMA-ScR (Preferred Reporting Items for Systematic Reviews and Meta-Analyses extension for Scoping Reviews) checklist (Multimedia Appendix 1).

### Information Sources, Eligibility Criteria, and Study Selection

We conducted literature searches in 5 databases—Ovid MEDLINE, Embase, CINAHL, Cochrane Library, and Web of Science—as well as Google Scholar. The search strategy was developed with the assistance of a librarian (Multimedia Appendix 2).

We included studies that described a telemedicine intervention for the management of hypertension, diabetes, or both; reported on the barriers to and facilitators of implementation; were published between 2017 and 2022 (as information and communication technologies evolve rapidly); and were published in English. We defined hypertension as persistently raised pressure in the blood vessels [41]. We defined diabetes as elevated levels of blood glucose, including type 1, type 2, and gestational diabetes [42]. Given our objective of exploring implementation barriers and facilitators, we did not restrict studies by implementation outcome. We excluded studies that did not meet the inclusion criteria or were editorials, commentaries, opinion pieces, or literature reviews. The eligibility criteria are listed in Textbox 1.

**Inclusion and exclusion criteria.**

- Inclusion criteria
  Describes a telemedicine intervention for the management of hypertension, diabetes, or both
  Reports on barriers to and facilitators of implementation of the intervention
  Published between 2017 and 2022
  Published in English
- Exclusion criteria
  Not focused on a telemedicine intervention for hypertension or diabetes management
  Study protocols and studies not reporting implementation facilitators and barriers
  Editorials, commentaries, opinion pieces, or literature reviews

The search results were imported into the Covidence software (Veritas Health Innovation Ltd) [43]. After duplicate removal, a 2-stage manual review process was conducted. In the first stage, a 3-member team (AK, QD, and EC) independently reviewed the titles and abstracts of the retrieved studies. The team then conducted a full-text review of eligible studies. In this stage, 2 team members (AK, QD, or EC) independently examined the full texts and excluded those that did not meet the eligibility criteria. Any disagreements were resolved via the third team member. The team conducted an additional full-text screening of the included studies to ensure that the eligibility criteria were strictly followed. Studies for which full-text reports could not be retrieved through web-based databases or library searches were excluded.

### Data Extraction and Synthesis

We extracted data including study characteristics (title, authors, publication year, country, aim, design, and population), intervention features (focus, duration, delivery, function, and setting), and barriers to and facilitators of implementation. The barriers to and facilitators of intervention implementation were extracted according to our modified working codebook (Multimedia Appendix 3), which was adapted from the CFIR construct codebook [44]. The coding results were analyzed iteratively using a deductive approach. The data from the included studies were tabulated, and a narrative synthesis was conducted. We summarized the common implementation barriers and facilitators across the studies according to the 5 major domains of the CFIR framework: intervention characteristics, outer setting, inner setting, characteristics of the individuals, and process.

## Results

### Search Results

**Figure 1:**
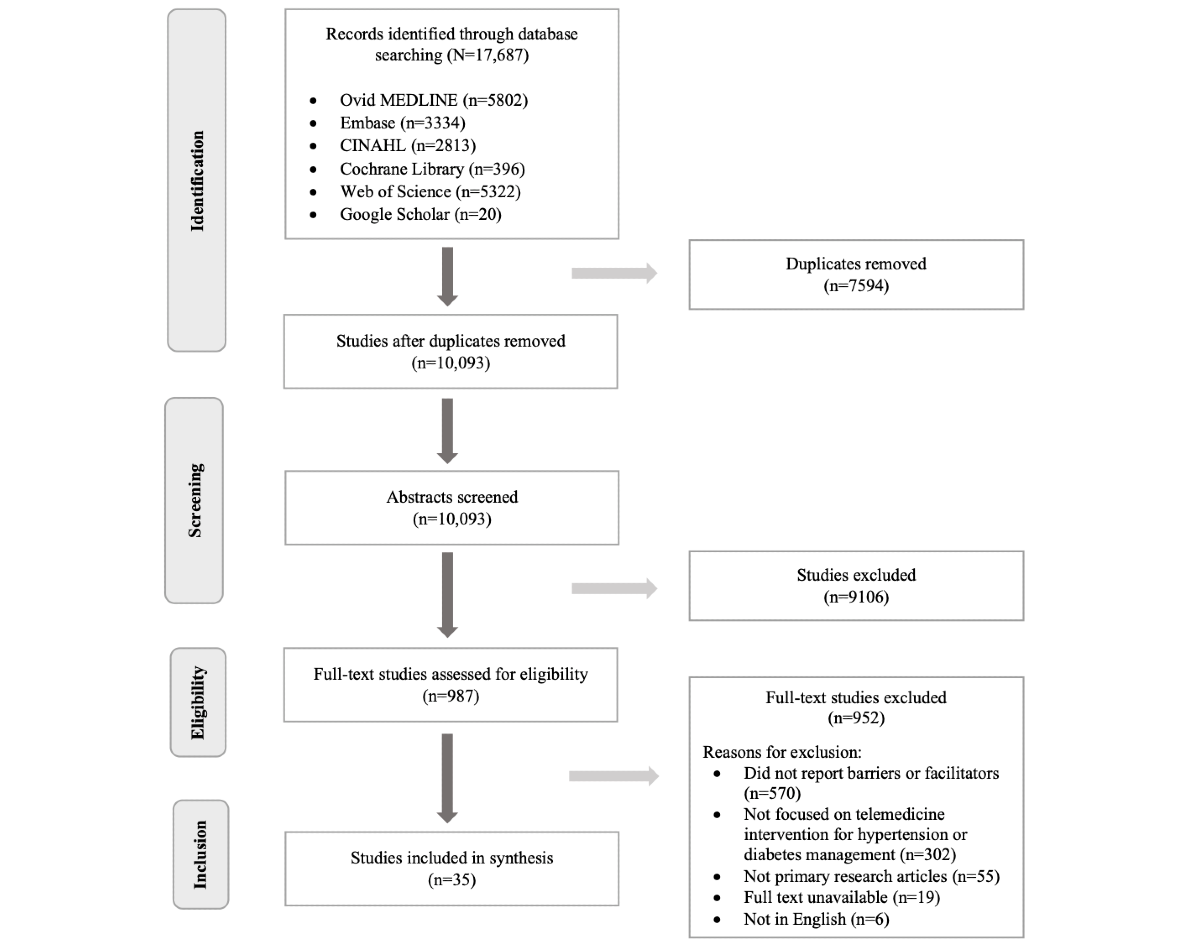
PRISMA (Preferred Reporting Items for Systematic Reviews and Meta-Analyses) flow diagram.

The initial search identified 17,687 articles, of which 7594 (42.94%) were removed as duplicates, and 9106 (90.22%) were then excluded based on title and abstract screening of 10,093 studies. We performed full-text screening of 987 articles, of which 952 (96.5%) did not meet our criteria, yielding 35 (3.5%) studies for inclusion (Figure 1).

### Study Characteristics

Table 1 offers a summary of the characteristics of the included studies. Detailed information is provided in Multimedia Appendix 4 [45-79]. Region and income groupings of countries were based on the World Bank 2022 classifications [80]. We found that almost all studies (32/35, 91%) were conducted in high-income countries, most of them (14/35, 40%) in the United States. Of the 35 studies, only 3 (9%) studies were conducted in low- and middle-income countries [45-47]. Most studies had qualitative designs (15/35, 43%), reported patient perspectives (20/35, 57%), and focused on diabetes care (29/35, 83%). A total of 46% (16/35) of the interventions were administered in the hospital, 20% (7/35) were administered in the community, and 14% (5/35) were administered in primary care settings. Most studies (18/35, 51%) aimed to support self-monitoring.

**Table 1:** Summary of characteristics of the studies included in the review (N=35).

Characteristics			Studies, n (%)
**Study region^a^**			
	North America (United States and Canada)	14 (40)	
	Europe and Central Asia (United Kingdom, Austria, Netherlands, Norway, Sweden, and Italy)	12 (34)	
	East Asia and the Pacific (Australia, Malaysia, and Cambodia)	4 (11)	
	Middle East and North Africa (Saudi Arabia)	3 (9)	
	Sub-Saharan Africa (Ethiopia)	2 (6)	
**Income level^a^**			
	High-income countries	32 (91)	
	Low- and middle-income countries	3 (9)	
**Study design**			
	Qualitative	15 (43)	
	Cross-sectional	7 (20)	
	Mixed methods	6 (17)	
	Randomized controlled trial	2 (6)	
	Other^b^	5 (14)	
**Reported perspective**			
	Patients only	20 (57)	
	Health care providers only	7 (20)	
	Both patients and health care providers	8 (23)	
**Disease focus of intervention**			
	Diabetes only	29 (83)	
	Hypertension only	2 (6)	
	Both diabetes and hypertension	4 (11)	
**Intervention modality^c^**			
	Smartphone app	13 (37)	
	SMS text messaging	10 (29)	
	Web-based	7 (20)	
	Phone call or voice messaging	7 (20)	
	Medical equipment (teleophthalmology or glucometer)	3 (9)	
**Aim of intervention**			
	Self-monitoring	18 (51)	
	Behavior change or education	11 (31)	
	Consultation	3 (9)	
	Other (tele-ophthalmological, medication adherence, or team-based care)	3 (9)	
**Administration of intervention**			
	Hospital	16 (46)	
	Community	7 (20)	
	Primary care clinic	5 (14)	
	University	3 (9)	
	Home-based	1 (3)	
	Not reported	3 (9)	

^a^Region and income groupings based on the World Bank 2022 classification.

^b^Other study designs included nonrandomized experimental studies, survey-based observational studies, case studies, and evaluations of implementation plans and processes.

^c^Sums to 40 studies because 4 studies used >1 modality to deliver their interventions.

### Telemedicine Interventions

The included studies reported several modalities for delivering interventions for hypertension or diabetes, including smartphone apps (13/35, 37%), SMS text messaging (10/35, 29%), web-based (7/35, 20%), phone calls or voice messaging (7/35, 20%), and medical equipment (3/35, 9%). We briefly describe each intervention modality in the following sections.

#### Smartphone Apps

A total of 37% (13/35) of the studies described telemedicine interventions involving smartphone apps. Garnweidner-Holme et al [48] examined the experiences of 9 HCP staff using the Pregnant+ app, which aimed to encourage behavior change for women with gestational diabetes in hospitals in Norway. The study concluded that the app was a useful tool for enhancing gestational diabetes care but that such apps should be culturally sensitive and technical problems must be addressed to ensure positive outcomes [48]. Vest et al [49] explored the perspectives of 8 nurses and administrators working for telemedicine vendors in a study on the implementation of app-based routine telemonitoring for patients with diabetes who are at high risk in a primary care setting in the United States. The findings emphasized the importance of integration and coordination between telemedicine agencies and health facilities as well as the role of telemedicine nurses in developing trust with patients [49]. Also in the United States, Yu et al [50] used a mixed methods approach to evaluate the acceptability of an app for self-monitoring among 118 Chinese and Hispanic immigrants with type 2 diabetes. The authors reported that the patient population would accept a mobile app for self-management but that their use of the app required consideration of their eyesight and the support of family in self-management [50].

In Ethiopia, Jemere et al [45] evaluated access to and willingness to use phone-based interventions among 423 patients with diabetes, including app components, for diabetes health services. Using a cross-sectional survey, the authors found that both access to a mobile phone and willingness to receive mobile phone–based health services were high in the study population [45]. Saiyed et al [51] described the rapid implementation of a comprehensive telehealth boot camp program for 37 patients with diabetes in the United States. The study reported on the success of a coordinated, team-based, and systematic approach with >100 patients enrolled, 75% of whom reported an improvement in their condition [51]. Breil et al [52] compared the acceptability of apps for self-monitoring of hypertension with usual care among 163 patients and 46 HCPs in Germany. Using the Unified Theory of Acceptance and Use of Technology, the authors explored predictors of intention to use the app [52]. Alshehri and Alshaikh [53] explored the implementation of an app for patients with prediabetes in Saudi Arabia using questionnaires with 48 patients and 20 HCPs. The study found that most patients thought the app would be useful for patients with prediabetes [53].

Alanzi [57], Desveaux et al [55], Du et al [54], and Bults et al [56] examined self-monitoring apps for type 2 diabetes management in Saudi Arabia, the United States, Canada, and the Netherlands, respectively. Alanzi [57] surveyed 33 HCPs to study obstacles to app implementation and reported several barriers to mobile health (mHealth) implementation in the region, ranging from limited mHealth expertise, funding, and infrastructure to organizational and bureaucratic concerns. Du et al [54] interviewed 10 patients with overweight or obesity about their experience with an app and found that, despite barriers, patients concluded that a technology-assisted self-monitoring intervention was beneficial, safe, and feasible. Using a qualitative realist evaluation approach with 16 participants, Desveaux et al [55] identified contextual factors that affect the usefulness of an app and reported how self-efficacy, competing priorities, previous behavior change, and beliefs about web-based solutions interact to determine engagement and affect clinical outcomes. Using mixed methods, Bults et al [56] analyzed quantitative data for 103 patients and qualitative data for 15 patients to understand the barriers to and drivers of app use. The authors reported on the importance of empowering HCP engagement and underscored the role of insurance companies in facilitating app use through reimbursements [56].

#### SMS Text Messaging

In total, 29% (10/35) of the studies described interventions involving SMS text messaging. Blair et al [58] implemented a 2-way SMS text messaging program (Text 4 Success) for 10 women with gestational diabetes. The authors reported that the program may be better suited for those who have low levels of adherence to self-monitoring blood glucose at baseline or at the time of their diagnosis of gestational diabetes [58]. Georgsson et al [59] implemented Care4Life, an interactive SMS text messaging service, among 10 patients with type 2 diabetes in the United States. The authors reported that the service filled the gap for longer-term use of mHealth systems in chronic disease management as patients were able to keep track of their disease and receive support during and between care visits [59]. Burner et al [60] and Avila-Garcia et al [61] examined the effect of family influence (n=24) and physical activity (n=26), respectively, on the use of SMS text messaging diabetes interventions among Latino patients with type 2 diabetes in the United States. These studies discussed the importance of culturally relevant programs to meet the needs of specific populations and that family members should be educated to provide effective social support [60,61]. Also in the United States, Horner et al [62] evaluated barriers to and facilitators of the Text to Move intervention, aimed at increasing physical activity using SMS text messaging and pedometers, among 46 patients with type 2 diabetes. Patients advocated for the personalization of texting frequency and for more contact time with HCPs to garner a stronger sense of support [62]. Rogers et al [63] evaluated barriers to and facilitators of implementing the Mobile Insulin Titration Intervention into usual care through interviews with 36 patients with type 2 diabetes and 19 HCPs in the United States. The patients and HCPs reported the intervention to be compatible with existing workflows and patients’ lifestyles but that initial implementation efforts should address staff training and nurse concerns [63].

Prinjha et al [64] and Bartlett et al [65] examined perceptions of medication adherence in the United Kingdom among 67 and 23 patients with type 2 diabetes, respectively. The authors discussed the importance of ensuring that SMS text messaging content is culturally relevant and novel [64,65]. Also in the United Kingdom, Grant et al [66] implemented a short SMS text messaging intervention for self-monitoring among 23 patients with type 2 diabetes. The authors noticed that SMS text messaging would be most beneficial if integrated into existing workflows [66]. Moreover, SMS text messaging was a component in the phone-based intervention by Jemere et al [45] and the pedometer intervention by Horner et al [62] for patients with diabetes.

#### Phone Call or Voice Messaging

A total of 20% (7/35) of the studies described telemedicine interventions involving phone calls or voice messaging. Jemere et al [45], as previously described, and Maietti et al [67] examined the individual and contextual determinants of diabetes interventions involving phone calls in Ethiopia and Italy, respectively. Maietti et al [67] surveyed 569 patients in the COVID-19 context and identified several sociodemographic factors that affected perceived quality and willingness to continue telemedicine services. The authors concluded that these factors should be considered in the implementation of care pathways integrating in-person visits with telemedicine services [67]. Al-Anezi [68] explored the readiness of 129 patients with hypertension or diabetes to adopt mobile phone interventions in Saudi Arabia. The study revealed that the population of Saudi Arabia is reluctant to adopt the eHealth system promoted in the Saudi Vision 2030 strategic plan [68]. Therefore, it is necessary to develop awareness campaigns to highlight the importance of eHealth and implement procedures to protect the confidentiality and security of patients’ medical records [68].

Timm et al [69] and Kobe et al [70] evaluated the implementation process of phone-based interventions. Timm et al [69] evaluated the fidelity of a phone-delivered health coaching intervention in Sweden to manage or prevent type 2 diabetes among 131 patients in relation to dimensions, enablers, and challenges. The authors found that tailoring interventions is necessary and language-skilled facilitators are needed to minimize barriers in intervention delivery [69]. Kobe et al [70] examined the implementation of Advanced Comprehensive Diabetes Care, an evidence-based phone call intervention for 230 patients with clinic-refractory, uncontrolled type 2 diabetes. The study found that, when strategically designed to leverage existing infrastructure, comprehensive telehealth interventions can be implemented successfully even in rural areas [70]. Steinman et al [47] evaluated the process of researchers partnering with a nongovernmental organization (MoPoTsyo) to implement a health behavior change voice messaging intervention aimed at improving NCD management for patients living with diabetes or hypertension in Cambodia through interviews with 20 patients and 6 HCPs. It was found that digital health alone is insufficient in countries with low-resource health systems and that high cell phone coverage did not translate to access [47]. Therefore, future digital health research and practice to improve NCD management in low- and middle-income countries requires engaging governments, nongovernmental organizations, and technology providers to work together to address barriers [47]. Finally, Brown-Johnson et al [71] explored the adoption and acceptability of team-based care involving telemedicine components, including phone calls, for patients with hypertension or diabetes via ethnography and interviews with 21 patients and 7 HCPs in the United States. The authors found that ethnography, conducted early in the implementation from a multistakeholder perspective, can provide rapid and actionable insights into where roles may need refinement or redefinition to support ultimate physical and mental health outcomes for patients [71].

#### Web-Based Interventions

A total of 20% (7/35) of the studies discussed interventions involving web-based components. Kolltveit et al [72] identified the perceptions of 34 HCPs in Norway on facilitators of engagement and participation in the application of an interactive web-based platform. The study found that successful larger-scale implementation of telemedicine must involve the consideration of complex contextual and organizational factors associated with different work settings [72]. Ross et al [73] investigated the barriers to and facilitators of implementing a web-based program in the United Kingdom by interviewing 34 HCPs. The authors concluded that, when planning and executing implementation activities in routine health care, of particular importance is the selection of an appropriate theory to guide the implementation process and selection of strategies, ensuring that enough attention is paid to planning implementation, and a flexible approach that allows for response to emerging barriers [73].

Muigg et al [74] analyzed the readiness of 47 Austrian patients with diabetes to avail web-based telemedicine and found that the top 3 barriers were data privacy issues, loss of personal communication and focus on blood sugar, and tele-physician competence. Seboka et al [46] assessed the readiness of 423 HCPs to use web-based telemonitoring technologies for managing patients with diabetes in Ethiopia. The study revealed that there was low awareness and readiness in participants regarding telemonitoring, although improving their attitudes, access to smartphones and computers, and technical skills may address readiness [46]. Similarly, Morton et al [75] explored 125 HCPs’ perceptions of implementing HOME BP, a web-based intervention aimed at reducing uncontrolled hypertension in primary care in the United Kingdom. The authors found that low trust in home readings and the decision to wait for more evidence influenced implementation for some practitioners, and contextual factors influencing implementation included the proximity of average readings to the target threshold [75]. Dening et al [76] described the development of a web-based dietary intervention based on the T2Diet Study for adults with type 2 diabetes in Australia through 21 patient interviews. The authors found that the relevance of resources, clear and simple positive communication, and flexibility for personal tailoring encouraged patients to engage [76]. Finally, the telehealth boot camp program by Saiyed et al [51] in the United States, as previously described, also included a web-based component.

#### Medical Equipment

A total of 9% (3/35) of the studies described telemedicine interventions involving medical equipment. Lee et al [77] explored the perspectives of 48 patients with type 2 diabetes mellitus who partook in a self-monitoring intervention using glucometers in Malaysia. They found that collaboration between educators, HCPs, telecommunication service providers, and patients is required to stimulate the adoption and use of telemedicine [77]. Liu et al [78] interviewed 20 patients and 9 HCPs to identify barriers to and facilitators of increasing teleophthalmology use in rural United States. The study found that patients and HCPs had limited familiarity with teleophthalmology for diabetic eye screening, and although HCPs were expected to initiate teleophthalmology referrals, they had considerable difficulty identifying when patients were due for screening [78]. In addition, the Text to Move intervention by Horner et al [62], as previously described, involved pedometers to monitor physical activity.

### Barriers and Facilitators

#### Overview

The barriers to and facilitators of implementing telemedicine interventions for hypertension or diabetes reported in the included studies are summarized in Table 2 and described in the following sections.

**Table 2:** Overview of Consolidated Framework for Implementation Research (CFIR) domains that were addressed in the studies as barriers to or facilitators of implementing telemedicine interventions for hypertension or diabetes (N=35).

CFIR framework constructs by domain		Barriers		Facilitators	
		Studies, n (%)	Specific barriers	Studies, n (%)	Specific facilitators
**Intervention characteristics**					
	Intervention source	N/A^a^	N/A	2 (6)	External expertise about clinical, operational, and telemedicine needs was sought, and new resources were added to support users.
	Relative advantage	1 (3)	There was uncertainty about whether all elements of the telemedicine intervention would be received, for example, SMS text messages.	7 (20)	Patients perceived the intervention as convenient, time-saving, timely, and practical for tracking physiological changes. Patients shared vision for the need for health care innovation. HCPs^b^ underwent training sessions for learning the procedures of the intervention.
	Adaptability	5 (14)	There was limited language availability and technical app issues.	7 (20)	The intervention was scalable, and patients were included in the development stages. The intervention included less costly components, such as SMS text messaging, and allowed for tailoring to diverse target populations.
	Trialability	N/A	N/A	2 (6)	Pilot clinics that were eager to innovate and overcome challenges were engaged.
	Complexity	6 (17)	The intervention required multiple steps to implement and orientation to nonroutine processes.	N/A	N/A
	Design quality and packaging	4 (11)	The intervention required time and attention to use, and components were repetitive or lacked interactivity. Digital components were not designed for older adult users.	12 (34)	The intervention was simple, user-friendly, and automated. Information and data were centralized in 1 place and easily visualized.
	Cost	9 (26)	Insurance did not cover telemedicine care fees, there were perceived marginal costs compared with in-person care, there was unwillingness to pay more for telemedicine services, or the initial infrastructure and maintenance costs were too high.	5 (14)	The intervention was cost-effective, used SMS text messaging, was free to participate in, and had financial aid options or a clear budget.
**Outer setting**					
	Patient needs and resources	8 (23)	There was lack of patient support from HCPs. Technical support was unavailable. Personal circumstances, such as culture, time, and rural settings, and the needs of disadvantaged groups, such as language barriers, were not accounted for.	12 (34)	Patients’ contextual and social environments, such as culturally relevant diet and exercise plans and family and friend engagement, were considered.
	Cosmopolitanism	N/A	N/A	1 (3)	There were interorganizational networks with local clinics, pharmacies, and laboratories.
	External policy and incentives	2 (6)	Cell phone networks were incompatible or had high messaging fees. There was limited Wi-Fi access in rural settings.	4 (11)	Public information campaigns were held for health care stakeholders. The environment supported healthy behaviors. There was alignment of intervention with international or national plans and earmarked government spending toward innovation in health or telemedicine.
**Inner setting**					
	Structural characteristics	4 (11)	Centralization of decision-making authority obstructed innovation. Telemedicine was a new service for the implementing organization. Bureaucracy, high turnover, rapid introduction of changes, and constraints on staff disrupted workflow.	3 (9)	Standards were set to ensure stability of HCPs and support the team, such as minimum workload and optimal fit with interactional workability, skill set workability, contextual integration, and relational integration. The implementing organization was credible as a mature and growing, horizontally structured organization.
	Networks and communications	5 (14)	There was weak intraorganizational information sharing about the intervention, especially across departments and hierarchical levels. Miscoordination across specialties introduced conflict in decisions about intervention protocol.	8 (23)	Caring relationships built on mutual trust were nurtured between patients and HCPs. There was consistent communication between HCPs, patients, implementers, and other stakeholders. Experts of different specialties (eg, network security) collaborated.
	Implementation climate	6 (17)	HCP buy-in and engagement was limited. Intervention objectives, workflows, and platforms were perceived as incompatible with existing professional scopes of practice and organizational processes. Telemedicine was not regarded as a priority or beneficial compared with existing work.	10 (29)	Intervention elements were contextualized with and embedded in existing organizational workflows, processes, and roles. The intervention was perceived as able to innovatively solve or reduce existing problems, such as reduce travel or wait times and improve convenience or ease of use.
	Readiness for implementation	6 (17)	There was a shortage of funding, staff, and expertise. There was a lack of access to digestible and credible information about the intervention and how to incorporate it into work tasks. Technological issues, such as software malfunctions and internet instability, could not be solved or circumvented.	5 (14)	A committed and responsible organizational leader supported conditions under which success of the intervention could be made possible. There were multiple methods for accessing credible and relevant information about the intervention for implementors and HCPs and about diabetes or hypertension for patients. Accessible and personalized dissemination of information (eg, training sessions) on use of telemedicine equipment for patients, as well as on-the-job training for HCPs, improved knowledge of and eased transition to the intervention.
**Characteristics of individuals**					
	Knowledge and beliefs about the intervention	6 (17)	Patients felt that meeting HCPs in person was a simpler and faster way to solve their queries than telemedicine. Patients did not trust web-based health care services and lacked understanding of diseases or telemedicine.	11 (31)	Patients believed technology to be supportive, convenient, encouraging, and helpful for their health and for keeping up with the era. Patients expressed awareness of added value of telemedicine as a tool for treating their conditions.
	Self-efficacy	5 (14)	Patients lacked self-motivation and self-discipline to adopt telemedicine and were reluctant to routinely record health behaviors.	10 (29)	Patients actively engaged with high empowerment to take control of their own health. Patients had a sense of accountability for their self-management and were confident in their ability to use telemedicine.
	Individual stage of change	1 (3)	Patients were frustrated with the episodic nature of managing their condition and with repeated unsuccessful attempts to “fine-tune” their self-management strategy.	6 (17)	Patients who were changing their treatment regimen, were newly diagnosed, were diagnosed with uncontrolled hypertension, or were not currently managing their blood pressure or blood glucose were more receptive to telemedicine. Improved health because of the intervention motivated further engagement and participation.
	Individual identification with organization	3 (9)	There were concerns about security and confidentiality of medical information, loss of face-to-face communication, and tele-physician proficiency.	2 (6)	Patients with previous, frequent interactions with HCPs and the implementing organization were more receptive to the intervention.
	Other personal attributes	12 (34)	Demographic factors or comorbidities influenced negative attitudes toward telemedicine, such as male sex, old age (>65 years), diabetic retinopathy, and side effect history. Differing personal priorities and issues influenced ability to partake in the intervention, such as lack of time, family pressure, and sharing phones with others.	11 (31)	Health literacy and technical literacy influenced positive attitudes toward telemedicine use, especially familiarity with technology, higher education level, and higher degree of innovativeness. Family involvement helped motivate participation.
**Process**					
	Planning	1 (3)	Input was not gathered from various stakeholders (eg, educators, HCPs, telecommunications service, and patients).	3 (9)	There was a lack of clarity about roles of team members, work culture, and patient involvement.
	Engaging	2 (6)	There was a lack of technical training and staff support that made it difficult to engage stakeholders.	6 (17)	Cohesive partnerships were built by engaging HCPs, champions, and other organizational networks.
	Executing	4 (11)	There was disagreement on the roles and responsibilities of staff in the implementation process.	1 (3)	There were frequent reminders of goals and scope and early goal setting and metrics for tracking the progress of the intervention.
	Reflecting and evaluating	2 (6)	There was a lack of opportunity for staff to receive patient feedback and reflect on the worth of the intervention.	5 (14)	There were opportunities for staff to reflect on the intervention through field-testing, user experience feedback, daily performance feedback, and synchronous interaction with other staff, especially when provided in relative rather than absolute terms.

^a^N/A: not applicable.

^b^HCP: health care provider.

#### Domain 1: Intervention Characteristics

The intervention characteristics domain focuses on the features of an intervention that might influence implementation [44]. A total of 60% (21/35) of the studies discussed factors that facilitated the implementation of telemedicine interventions for hypertension or diabetes [47,49,51,54,59-63,65-67,70-76,79]. In total, 29% (2/7) of the web-based studies reported that positive perceptions of externally developed telemedicine innovation, described as gathering external expertise on clinical, operational, and telemedicine areas, as well as adding new resources to support users may influence success of implementation [51,76]. A total of 20% (7/35) of the studies reported that patients perceived convenience, timeliness, and practicality for tracking blood pressure or blood sugar changes to be advantages for implementing telemedicine interventions [47,51,63,66,70,73,75]. HCPs perceived telemedicine to be more advantageous than regular care if they underwent training sessions for learning the procedures of the intervention [63,73]. The intervention was deemed to be easily adaptable to meet local needs if it was scalable, involved patients in its development, and included cost-saving components such as SMS text messaging [47,49,51,59,60,65,71]. Interventions that were packaged in a simple and user-friendly way, centralized information in 1 place, automated processes such as calculations, and easily visualized data trends were regarded as well designed [51,54,67,79]. In addition, interventions that offered free participation, had financial aid options, were cost-effective, had a clear budget, and used existing infrastructures were reported to have more successful implementation [51,54,59,61-63,65-67,70,74,75,78,79].

Barriers to intervention implementation included patient uncertainty about whether all elements of the intervention, such as SMS text messages, were being received [66], as well as limited language availability [63] and app-related technical issues [47,66,79]. A total of 17% (6/35) of the studies reported complexity-related barriers, including when the intervention required log-in to multiple websites [47,48,56,65,66,79], when data entry and importing were not straightforward [47,66], and when there was a steep learning curve to understanding telemedicine components [56]. Interventions were deemed to be poorly designed for implementation when they required time and attention to use [66,72], when components such as SMS text messages were repetitive or lacked interactivity [62], and when components were not designed with older adult users in mind [59]. Moreover, 26% (9/35) of the studies reported that costs affected telemedicine implementation if insurance did not cover telemedicine care fees [56,78], when there were perceived marginal costs compared with in-person care [77], when there was an unwillingness to pay more for telemedicine services [74], and when initial infrastructure and maintenance costs were too high [47,57,79].

#### Domain 2: Outer Setting

The outer setting domain includes features of the external context or environment, such as the economic, political, and social contexts, that might influence intervention implementation. The most reported construct in this domain among the included studies was patient needs and resources, with 34% (12/35) of the studies reporting facilitators and 23% (8/35) reporting barriers. Patient needs and resources refers to the extent to which patient needs are known and prioritized by the organization [44]. Intervention implementation was successful if it included personalized information, provided discussion forums, ensured patients’ convenience in accessing care, integrated culturally relevant diet and exercise plans, and leveraged family or friend engagement [45,47,48,50,51,53,59,60,65,67,68,78]. In contrast, intervention implementation was difficult when there was a lack of patient support from HCPs; technical support was unavailable; and linguistic, cultural, or transportation needs of individuals, especially disadvantaged groups, were unmet [47,48,50,56,62,68,77,79].

Only 3% (1/35) of the studies reported a facilitating factor in the cosmopolitanism construct. Cosmopolitanism refers to the degree to which an organization is networked with other external organizations [44]. Networks between the implementing organization and local clinics, pharmacies, and laboratories made it more likely to implement new telemedicine initiatives quickly [47].

A total of 17% (6/35) of the studies reported barriers and facilitators in the external policy and incentives construct, which refers to external strategies to spread interventions [44]. Facilitators included the presence of public information campaigns for health care stakeholders, environments supportive of healthy behaviors, alignment of the intervention with international or national plans, and earmarked government spending toward innovation in health or telemedicine [47,68,74,76]. Barriers included a lack of legal and regulatory policies that support telemedicine, such as incompatible cell phone networks with high messaging fees and limited Wi-Fi access in rural settings [47,57].

#### Domain 3: Inner Setting

The inner setting domain encompasses the structural, cultural, and political contexts within the organization that might influence the implementation of interventions. A total of 17% (6/35) of the studies reported barriers and facilitators in the structural characteristics construct, which refers to the environment, including factors such as age, architecture, maturity, and size of the organization, where the intervention is conducted [44]. Ross et al [73] found that the novelty of telemedicine was a barrier to its implementation in more mature organizations. In contrast, Steinman et al [47] found that organizations’ credibility as mature, growing, and horizontally structured was conducive to the implementation of a telemedicine intervention. Most studies reporting on structural characteristics (4/7, 57%) agreed that unstable teams, which have high turnover, rapid introduction of changes, constraints on staff, and bureaucratic environments, disrupted workflow [57,62,73,77], whereas standards that ensured the stability of teams, such as minimum workload and optimal fit with interactional and skill set workability, facilitated the success of implementation [67,71,73].

The networks and communications construct, which encompasses the social networks and communications within the organization [44], was discussed in 23% (8/35) of the studies. Miscoordination and weak intraorganizational information sharing introduced conflict and obstructed intervention implementation [51,72,73,78]. Ensuring positive relationships, including consistent communication between stakeholders, trust between patients and HCPs, and collaboration between experts of different specialties, helped overcome challenges to intervention implementation [49,70,72,77].

The implementation climate construct refers to the extent to which an intervention is supported and expected within an organization [44] and was discussed in 31% (11/35) of the studies. Limited HCP buy-in, perceived incompatibility of the intervention with existing organizational workflows and professional scopes of practice, and perceived unimportance of telemedicine were found to reduce the receptivity of interventions [49,51,63,67]. Alternatively, interventions that were contextualized and embedded within existing organizational workflows and roles were supported [63,67,75]. A perception that the intervention was innovative and able to solve existing problems, such as long wait times, was also important for successful intervention implementation [47,51,54,70,74,77].

A total of 29% (10/35) of the studies described readiness for implementation, which refers to tangible indicators of an organization’s commitment to their decision to implement an intervention [44]. A shortage of funding, staff, and expertise was the most reported barrier to the implementation of a telemedicine intervention in this construct [57,70]. Horner et al [62] and Lee et al [77] also reported the inability to work around technological issues, such as internet instability, as a barrier to implementation. Furthermore, a lack of access to digestible information about the intervention as well as how to incorporate the intervention into work tasks was highlighted by multiple studies (6/11, 55%) [49,56,65,72,76,78]. Multiple methods for accessing credible and relevant information about the intervention for implementors and about diabetes or hypertension for patients were key for successful implementation [56,77,78]. Personalized information about the appropriate use of telemedicine equipment, delivered through personalized means such as training sessions incorporated into the intervention, helped improve patient knowledge and acceptance of the intervention [74,78]. For HCPs, on-the-job training was conducive to them feeling well informed and eased any transition required for the intervention [51]. Finally, Horner et al [62] found that a committed and responsible leader who is able to monitor and support the conditions required for the intervention to be possible would help overcome many organizational challenges.

#### Domain 4: Characteristics of Individuals

The characteristics of individuals domain refers to the factors related to the individuals involved in intervention implementation. The knowledge and beliefs about the intervention construct encompasses individual attitudes toward and value placed on the intervention [44]. Knowledge and beliefs about the intervention were reported as an implementation barrier in 17% (6/35) of the studies. Some patients believed that meeting their HCPs in person was a simpler and faster way to solve their queries than telemedicine [77]. In addition, some patients did not trust web-based health care services and lacked an understanding of telemedicine and diseases [60,68,69,78,79]. In contrast, 31% (11/35) of the studies reported that patients believed that using technology was supportive, convenient, encouraging, and helpful for their health and for keeping up with the times [46,50,53,55,59,63,67,74].

A total of 14% (5/35) of the studies identified low self-efficacy as a barrier to implementation as patients lacked the self-motivation and self-discipline to adopt telemedicine and were reluctant to routinely record health behaviors [50,54,61,65,66]. In contrast, 29% (10/35) of the studies indicated that self-efficacy could be a facilitator. Some patients actively felt empowered to take control of their own blood pressure or blood glucose levels, whereas others had a sense of accountability and were confident in their ability to use technology for health [53,55,63-66,68,70,75,77].

The individual stage of change construct was addressed as a barrier and as a facilitator of implementing telemedicine interventions in 3% (1/35) and 17% (6/35) of the studies, respectively. The individual stage of change construct refers to the phase an individual is in as they progress toward the use of the intervention [44]. In total, 3% (1/35) of the studies showed patients’ frustrations with the episodic nature of managing their condition and repeated unsuccessful attempts to “fine-tune” their self-management strategy [55]. In contrast, patients who were changing their treatment regimen, were newly diagnosed, were diagnosed with uncontrolled hypertension, or were not currently managing their hypertension or diabetes were more inclined to adopt telemedicine [47,49,51,55,65]. In addition, the result of improved health in the process of implementation often helped motivate patients to continue participating in the intervention [63].

In total, 14% (5/35) of the studies discussed individual identification with an organization, which refers to how individuals perceive the organization and their relationship and degree of commitment to that organization [44]. Of these 5 studies, 3 (60%) reported patient concerns about the security and confidentiality of medical information, loss of face-to-face communication, and uncertainty about tele-physician proficiency as barriers to intervention implementation [68,74,77]. The other 40% (2/5) of the studies showed that patients with previous, frequent interactions with HCPs and the implementing organization were more receptive to the intervention [47,74].

Patients’ personal attributes were often highlighted as barriers to or facilitators of intervention implementation in the included studies. Consideration of demographic factors was important for ensuring the success of telemedicine. Male patients and patients aged >65 years were more likely to have difficulty with self-motivation than female counterparts [54,59,67,77,79]. Lack of time, family pressure, and sharing phones with others were also negatively associated with intervention implementation [54,65]. Furthermore, among patients with hypertension, those taking medication in the form of a pill were more willing to receive mobile phone–based health services than those taking injectable insulin [45]. Patients’ multiple medication or side effect history ruled out many potential telemedicine opportunities because of concerns about the complexity of treatment and patient anxiety about the adequacy of treatment [65,75]. Among patients with type 2 diabetes, those with diabetic retinopathy were less likely to adopt telemedicine as they often found it hard to use their phones or the internet [50]. In contrast, factors that engendered a positive attitude toward telemedicine included high health and technical literacy, higher education, unemployment, and family involvement and motivation [45,46,59,60,67,74,77].

#### Domain 5: Process

The process domain includes strategies for planning, engaging, executing, and reflecting on and evaluating the implementation of an intervention [44]. At the planning stage, failure to elicit input from various stakeholders, including educators, HCPs, telecommunications service providers, and patients, made it challenging to successfully implement telemedicine interventions [77]. In contrast, clarity about the roles of team members, work culture, and patient involvement facilitated implementation [48,61,71].

At the engagement stage, 6% (2/35) of the studies reported a lack of technical telemedicine training and staff support as barriers [68,73]. Al-Anezi [68] described how a lack of staff support was an issue when efforts were needed to integrate the intervention into practice. A total of 17% (6/35) of the studies found that overall support from executives and HCPs, staff’s ease of access to information about the intervention, formal and informal networks to communicate about the intervention, the presence of a strong champion leader, leveraging partnerships, and understanding and accounting for local needs were facilitators of intervention implementation [48,51,63,70,71,77].

A total of 14% (5/35) of the studies reported barriers and facilitators at the execution stage. Barriers included a lack of agreement on the roles and responsibilities of staff in the implementation process, inconsistent reminder systems, interfacilitator variability in delivering the intervention, and lack of direct computer access [69,71,73,78]. In contrast, Saiyed et al [51] reported that setting early goals and metrics for the intervention and setting frequent reminders about intervention goals facilitated execution.

In total, 17% (6/35) of the studies reported facilitators and barriers at the reflection and evaluation stage. Barriers were related to a lack of opportunity for staff to receive patient feedback and reflect on the worth of the intervention [66,73]. In contrast, staff participation could be increased if there were more opportunities to reflect on the relative worth of the intervention through feedback from patients, such as through field-testing or user experience feedback, as well as with synchronous interaction with other staff [48,61,66,70,76].

## Discussion

### Principal Findings

This scoping review used a comprehensive implementation science framework to identify the barriers to and facilitators of implementing telemedicine interventions for hypertension or diabetes care. Our findings provide important insights into the factors that should be considered to improve telemedicine implementation for patients with diabetes, hypertension, or both. Within the intervention characteristics domain, interventions that were designed in a simple and user-friendly manner and centralized automated data most frequently facilitated successful implementation. In contrast, interventions that were costly for the implementing organization or patients were difficult to implement. In the outer setting domain, telemedicine interventions were commonly implemented successfully if they considered patients’ contextual and social environments, such as by being culturally competent. Interventions that did not account for patients’ needs, especially language or technological skills, were difficult to implement. Regarding the inner setting domain, it was important for telemedicine interventions to be compatible with and embedded within existing organizational goals and workflows. Lack of knowledge of how to incorporate the intervention into workflows or goals hindered implementation. Within the characteristics of individuals domain, technical literacy, the perception that technology was convenient, and family involvement supported telemedicine intervention implementation, whereas competing priorities, comorbidities, and older age were barriers. Finally, in the process domain, engaging organizational networks to build cohesive partnerships and opportunities for feedback most commonly facilitated intervention implementation. A lack of training and agreement on staff responsibilities hindered the implementation process.

Our results are similar to those of recent reviews examining the implementation of telemedicine interventions. For instance, Dovigi et al [81], Betancourt et al [82], and Whitelaw et al [83] found user-friendliness and cost concerns to be the most common factors affecting implementation. Whitelaw et al [83] also found that training and integration with existing workflows are important for successful telemedicine implementation. Perceptions of technology and social support were identified by Kruse and Heinemann [31] as key factors facilitating the implementation of telemedicine interventions. Schreiweis et al [84] further found that agreement between goals helped ensure that the intervention was regarded as a priority and beneficial by all stakeholders, which is critical for successful implementation. However, most barriers and facilitators to telemedicine implementation identified in recent reviews were within the intervention characteristics and characteristics of individuals domains [31,81]. Very little information was reported within the outer setting, inner setting, and process domains [82-84]. In contrast, the 5 CFIR domains were addressed almost equally in our review.

As the burden of NCDs grows and more people in resource-constrained health systems require ongoing care, people-centered telemedicine will be an increasingly important means of accessing health services. Indeed, the World Health Organization global strategy on integrated people-centered health services describes how telemedicine, when properly implemented, can be a powerful tool for equitable care, able to reach even the most marginalized communities [85]. However, not all telemedicine is currently able to reach those most in need. The COVID-19 pandemic has demonstrated how emergencies can lead to rapid but often uncoordinated and inequitable implementation of telemedicine for NCD care [86,87]. Successful implementation requires not only technological innovation but also an understanding of both the user and their context across multiple dimensions. Therefore, through the lens of the CFIR and according to our findings, we propose several key recommendations and provide examples for successful planning, engagement, execution, and reflection and evaluation stages when implementing a telemedicine intervention for hypertension, diabetes, or both (Table 3) [88].

First, an effective telemedicine intervention for hypertension or diabetes needs a user-friendly design with flexibility to tailor to both user and contextual needs [89]. The implementor should engage diverse stakeholders and sources of expertise within or outside the implementing organization, such as those with knowledge of network and information security, to improve each element of the intervention. Adequate financial investment at the planning stage of implementation can help ensure that the rest of the intervention is cost-effective and prevent any missteps. The goal of the intervention should be consistent with organizational goals and meet local needs.

Second, continuous collaboration with stakeholders involved in the intervention, including staff, patients, clinics, pharmacies, laboratories, and communities, is an ideal approach to maintaining an efficient and motivated implementation process. In addition, family and peer supports are of great value to motivate participation in interventions. This is in line with existing reviews that suggest that overall support from HCPs, executives, and patients’ social networks improves performance and acceptance during implementation [31,82].

Third, it is vital to keep the process of decision-making, problem-solving, and collaborating systematic and thorough when executing the implementation. Barriers to implementation emerge with uncertainty or a lack of knowledge of the intervention and its implementation context. For instance, some telemedicine interventions were interrupted and even cancelled owing to weak Wi-Fi connection in low-resource areas [90]. This negatively affected stakeholders’ perception of the intervention.

Finally, robust evaluation and reflection are important for the success of an intervention. Regular and synchronous performance and experience feedback from patients helps staff better understand patients’ needs in real time and allows staff to reflect on the worth of the intervention. A recent study indicated that feedback from diverse patient groups is especially important as it enables the implementation of a more inclusive and adaptable intervention [83]. Groups may include patients with suspected hypertension, older adults, medically underserved people, high-risk patients with diabetes, patients with comorbidities, and patients isolated because of pandemics or other disasters [83].

**Table 3:** Summary of recommendations and examples of success for facilitating implementation of telemedicine interventions for diabetes or hypertension.

Stage of intervention implementation	Recommendations for success	Examples of success
Planning	Design user-friendly intervention with clear instructions and easily visualizable information for HCPs^a^ and patients. Engage stakeholders with expertise in clinical, operational, organizational, interorganizational, and telemedicine domains. Introduce financial aid schemes to ensure that participants do not face cost-related barriers. Align incentives and intervention goals across stakeholders and organizational, national, or global efforts to increase buy-in and access to earmarked funding and staff.	Familiar SMS text messaging features were used to deliver the intervention [63]. Strong leadership was identified at implementation sites, and there was collaboration between sites and with key government stakeholders [70]. SMS text messaging was a scalable and cost-effective method of facilitating communication between patients and HCPs [62]. Compatibility of intervention with clinic operations incentivized HCP involvement [63].
Engaging	Provide technical training to both HCPs and patients. Ensure consistent communication for relationship of mutual trust between patients and HCPs. Encourage family involvement and peer support.	Mock previsit training for patients and remote training for HCPs enhanced acceptance and preparedness [51]. Continuity in relationship through consistent communication enabled HCPs to generate trust, uncover social and economic factors affecting patients, and provide a sense of security [49]. Family assisted and encouraged patients to use technology [54].
Executing	Maintain high-standard and high-quality systems for decision-making, problem-solving, and collaboration. Consider security and confidentiality of medical information of patients.	Intentional site selection, use of existing and effective infrastructure, site-specific adaptations, coordination and communication across sites, and a mentored approach were involved in implementation [70]. Patient perception of data privacy and security is important for acceptance of the intervention [54].
Reflecting and evaluating	Collect regular and synchronous performance and user experience feedback.	Multiple opportunities to incorporate user feedback contributed to low rates of patient dropout [76].

^a^HCP: health care provider.

### Strengths and Limitations

To our knowledge, our review is the first to use an implementation science framework to explore barriers to and facilitators of implementing telemedicine interventions for managing diabetes, hypertension, or both. A key strength of our review was the use of a comprehensive implementation research framework to guide the data collection, analysis, and synthesis as this ensured that we addressed our research question comprehensively and systematically.

A limitation of our review was that we excluded non-English studies and gray literature from our search. The rapid adoption of telemedicine interventions in recent years may have been captured more quickly in gray literature. Therefore, we may have missed potentially relevant studies. In addition, assessing implementation barriers and facilitators was not the primary aim of all the included studies. However, we used discussion and consensus to identify and classify barriers and facilitators.

### Conclusions

This scoping review used a comprehensive implementation research framework to synthesize the barriers to and facilitators of implementing telemedicine interventions for hypertension or diabetes care. Our findings and recommendations highlight that successful intervention implementation needs comprehensive efforts to overcome challenges from the individual and interpersonal to the organizational and environmental levels. The needs and perceptions of patients, HCPs, and other staff must be prioritized and accommodated, including technical and health literacy, roles and responsibilities, and information sharing. Communication between patients and HCPs as well as partnerships with different experts are important at the interpersonal level. Embedding intervention goals and processes within existing organizational goals and workflows is important for engaging stakeholders, facilitating collaboration, and ensuring HCP buy-in. Finally, regulatory and legal flexibility, supportive environments, and alignment of the intervention with national policies are key to ensuring funding, staff, and overall successful implementation.

## Appendix

Multimedia Appendix 1 PRISMA-ScR (Preferred Reporting Items for Systematic Reviews and Meta-Analyses extension for Scoping Reviews) checklist.

Multimedia Appendix 2 Detailed search strategies.

Multimedia Appendix 3 Consolidated Framework for Implementation Research codebook.

Multimedia Appendix 4 Detailed study characteristics.

## Abbreviations

CFIR: Consolidated Framework for Implementation Research
HCP: health care provider
mHealth: mobile health
NCD: noncommunicable disease
PRISMA-ScR: Preferred Reporting Items for Systematic Reviews and Meta-Analyses extension for Scoping Reviews
