# Supplementary material for: Implementation Science Perspectives on Implementing Telemedicine Interventions for Hypertension or Diabetes Management: Scoping Review
Source: J Med Internet Res. 2023 Mar 14;25:e42134. doi: 10.2196/42134 (PMC10131907; doi:10.2196/42134)
Supplement: Multimedia Appendix 2 [file jmir_v25i1e42134_app2.doc]

**Multimedia Appendix 2. Detailed search strategies.**

Ovid MEDLINE

April 25, 2022

1 exp Hypertension/ or exp Blood pressure/ 524979

2 (hypertens* or prehypertens* or "high blood pressure" or HTN or HBP).tw,kf. 493340

3 ((blood or arterial or diastolic or systolic) adj3 pressure).tw,kf. 402750

4 ((elevat$ or increas$ or high or rais$ or rising) adj2 (bp or dbp or hbp or sbp)).tw,kf. 13655

5 1 or 2 or 3 or 4 891335

6 diabetes mellitus/ or diabetes mellitus, type 1/ or diabetes mellitus, type 2/ or exp Diabetes Mellitus, Type 1/ or exp Diabetes Mellitus, Type 2/ 346784

7 (diabet* or prediabet* or "blood sugar" or "diabetes mellitus" or "type 1 diabetes" or "type 2 diabetes" or "type 1 diabetes mellitus" or "type 2 diabetes mellitus" or T1D or T2D or T1DM or T2DM or "non insulin* depend*" or "noninsulin* depend*" or "noninsulindepend*" or "non insulin?depend*" or NIDDM or "insulin* depend*" or "insulindepend*" or "insulin?depend*" or IDDM).tw,kf. 733139

8 6 or 7 771328

9 5 or 8 1525300

10 exp Telemedicine/ or Distance Counseling/ or Remote Consultation/ 40081

11 (telemed* or teleadminist* or teleadvice or teleassess* or telecare or telechat* or teleconf* or teleconsult* or telecounsel* or teledeliv* or telehealth* or teleinterv* or telemanag* or telemonit* or telenurs* or telepharm* or televisit* or teletherap* or videochat* or videotelephon*).tw,kf. 33508

12 (eConsult* or e-consult* or ecounsel* or e-counsel* or eHealth* or e-Health* or einterv* or e-interv* or etherap* or e-therap* or mHealth* or m-Health* or mobile health*).tw,kf. 21422

13 ((comput* or distance or internet or phone or online or remote or tele* or video or virtual or web) adj2 (administ* or advice or assess* or care or chat* or confer* or consult* or counsel* or deliver* or health* or interv* or manag* or medic* or monit* or nurs* or pharm* or therap* or visit*)).tw,kf. 127628

14 (App or apps or facetime* or helpline* or store-and-forward* or store-forward* or skype* or video* or zoom or "webbased tool" or "web-based tool*" or voice-over or voiceover or VoIP).tw,kf. 193750

15 10 or 11 or 12 or 13 or 14 342978

16 Health plan implementation/ or program evaluation/ 72271

17 (intervention* or program* or develop* or implement* or campaign or evaluat* or assess* or pilot or impact).tw,kf. 11642500

18 16 or 17 11651149

19 9 and 15 and 18 12320

20 limit 19 to (english language and yr="2017 -Current") 5802

EMBASE

April 25, 2022

(exp AND hypertension^ OR exp) AND blood AND pressure^ 25093

hypertens* OR prehypertens* OR 'high blood pressure' OR htn OR hbp 1160522

(blood OR arterial OR diastolic OR systolic) AND pressure 962146

((elevat$ or increas$ or high or rais$ or rising) (bp or dbp or hbp or sbp)) 130463

#1 OR #2 OR #3 OR #4 1825904

(((diabetes AND mellitus^ OR diabetes) AND 1^or AND diabetes AND mellitus, AND type AND 2^ OR exp) AND diabetes AND mellitus, AND type AND 1^ OR exp) AND diabetes AND mellitus, AND type AND 2^ 12671

diabet* OR prediabet* OR 'blood sugar' OR 'diabetes mellitus' OR 'type 1 diabetes' OR 'type 2 diabetes' OR 'type 1 diabetes mellitus' OR 'type 2 diabetes mellitus' OR t1d OR t2d OR t1dm OR t2dm OR 'non insulin* depend*' OR 'noninsulin* depend*' OR 'noninsulindepend*' OR 'non insulin?depend*' OR niddm OR 'insulin* depend*' OR 'insulindepend*' OR iddm 1493236

#6 OR #7 1493236

#5 OR #8 2943373

((exp AND telemedicine^ OR distance) AND counseling^ OR remote) AND consultation^ 3682

telemed* OR teleadminist* OR teleadvice OR teleassess* OR telecare OR telechat* OR teleconf* OR teleconsult* OR telecounsel* OR teledeliv* OR telehealth* OR teleinterv* OR telemanag* OR telemonit* OR telenurs* OR telepharm* OR televisit* OR teletherap* OR videochat* OR videotelephon* 2966

(econsult* OR 'e consult*' OR ecounsel* OR 'e counsel*' OR ehealth* OR 'e health*' OR einterv* OR 'e interv*' OR etherap* OR 'e therap*' OR mhealth* OR 'm health*' OR mobile) AND health* 91084

(comput* OR distance OR internet OR phone OR online OR remote OR tele* OR video OR virtual OR web) AND adj2 AND (administ* OR advice OR assess* OR care OR chat* OR confer* OR consult* OR counsel* OR deliver* OR health* OR interv* OR manag* OR medic* OR monit* OR nurs* OR pharm* OR therap* OR visit*) 5

app OR apps OR facetime* OR helpline* OR 'store and forward*' OR 'store forward*' OR skype* OR video* OR zoom OR 'webbased tool' OR 'web-based tool*' OR 'voice over' OR voiceover OR voip 317900

#10 OR #11 OR #12 OR #13 OR #14 452100

(health AND plan AND implementation^ OR program) AND evaluation^ 184528

intervention* OR program* OR develop* OR implement* OR campaign OR evaluat* OR assess* OR pilot OR impact 17781369

#16 OR #17 17781369

#9 AND #15 AND #18 22865

#19 AND (2017:py OR 2018:py OR 2019:py OR 2020:py OR 2021:py OR 2022:py) AND 'human'/de AND 'article'/it

#19 AND (2017:py OR 2018:py OR 2019:py OR 2020:py OR 2021:py OR 2022:py) AND 'human'/de AND 'article'/it AND ('diabetes mellitus'/dm OR 'diabetic foot'/dm OR 'diabetic retinopathy'/dm OR 'hyperglycemia'/dm OR 'hypertension'/dm OR 'insulin dependent diabetes mellitus'/dm OR 'non insulin dependent diabetes mellitus'/dm) 3334

EBSCOhost CINAHL

April 25, 2022

| **#** | Query | Limiters/Expanders | Results |
| --- | --- | --- | --- |
| S20 | S9 AND S15 AND S18 | Limiters - Published Date: 20170101-20221231 Narrow by Language: - english Search modes - Boolean/Phrase | 2,813 |
| S19 | S9 AND S15 AND S18 | Search modes - Boolean/Phrase | 6,084 |
| S18 | S16 OR S17 | Search modes - Boolean/Phrase | 2,775,364 |
| S17 | TI ( (intervention* or program* or develop* or implement* or campaign or evaluat* or assess* or pilot or impact) ) OR AB ( (intervention* or program* or develop* or implement* or campaign or evaluat* or assess* or pilot or impact) ) | Search modes - Boolean/Phrase | 2,755,240 |
| S16 | (MH "Program Development+") | Search modes - Boolean/Phrase | 93,128 |
| S15 | S10 OR S11 OR S12 OR S13 OR S14 | Search modes - Boolean/Phrase | 170,125 |
| S14 | TI ( (App or apps or facetime* or helpline* or store-and-forward* or store-forward* or skype* or video* or zoom or "webbased tool" or "web-based tool*" or voice-over or voiceover or VoIP) ) OR AB ( (App or apps or facetime* or helpline* or store-and-forward* or store-forward* or skype* or video* or zoom or "webbased tool" or "web-based tool*" or voice-over or voiceover or VoIP) ) | Search modes - Boolean/Phrase | 63,980 |
| S13 | TI ( ((comput* or distance or internet or phone or online or remote or tele* or video or virtual or web) N2 (administ* or advice or assess* or care or chat* or confer* or consult* or counsel* or deliver* or health* or interv* or manag* or medic* or monit* or nurs* or pharm* or therap* or visit*)) ) OR AB ( ((comput* or distance or internet or phone or online or remote or tele* or video or virtual or web) N2 (administ* or advice or assess* or care or chat* or confer* or consult* or counsel* or deliver* or health* or interv* or manag* or medic* or monit* or nurs* or pharm* or therap* or visit*)) ) | Search modes - Boolean/Phrase | 91,087 |
| S12 | TI ( (eConsult* or e-consult* or ecounsel* or e-counsel* or eHealth* or e-Health* or einterv* or e-interv* or etherap* or e-therap* or mHealth* or m-Health* or mobile health*) ) OR AB ( (eConsult* or e-consult* or ecounsel* or e-counsel* or eHealth* or e-Health* or einterv* or e-interv* or etherap* or e-therap* or mHealth* or m-Health* or mobile health*) ) | Search modes - Boolean/Phrase | 9,462 |
| S11 | TI ( (telemed* or teleadminist* or teleadvice or teleassess* or telecare or telechat* or teleconf* or teleconsult* or telecounsel* or teledeliv* or telehealth* or teleinterv* or telemanag* or telemonit* or telenurs* or telepharm* or televisit* or teletherap* or videochat* or videotelephon*) ) OR AB ( (telemed* or teleadminist* or teleadvice or teleassess* or telecare or telechat* or teleconf* or teleconsult* or telecounsel* or teledeliv* or telehealth* or teleinterv* or telemanag* or telemonit* or telenurs* or telepharm* or televisit* or teletherap* or videochat* or videotelephon*) ) | Search modes - Boolean/Phrase | 16,028 |
| S10 | (MH "Telehealth") OR (MH "Telemedicine+") OR (MH "Telenursing") | Search modes - Boolean/Phrase | 31,015 |
| S9 | S5 OR S8 | Search modes - Boolean/Phrase | 425,727 |
| S8 | S6 OR S7 | Search modes - Boolean/Phrase | 262,011 |
| S7 | TI ( (diabet* or prediabet* or "blood sugar" or "diabetes mellitus" or "type 1 diabetes" or "type 2 diabetes" or "type 1 diabetes mellitus" or "type 2 diabetes mellitus" or T1D or T2D or T1DM or T2DM or "non insulin* depend*" or "noninsulin* depend*" or "noninsulindepend*" or "non insulin?depend*" or NIDDM or "insulin* depend*" or "insulindepend*" or "insulin?depend*" or IDDM) ) OR AB ( (diabet* or prediabet* or "blood sugar" or "diabetes mellitus" or "type 1 diabetes" or "type 2 diabetes" or "type 1 diabetes mellitus" or "type 2 diabetes mellitus" or T1D or T2D or T1DM or T2DM or "non insulin* depend*" or "noninsulin* depend*" or "noninsulindepend*" or "non insulin?depend*" or NIDDM or "insulin* depend*" or "insulindepend*" or "insulin?depend*" or IDDM) ) | Search modes - Boolean/Phrase | 232,438 |
| S6 | (MH "Diabetes Mellitus") OR (MH "Diabetes Mellitus, Type 1+") OR (MH "Diabetes Mellitus, Type 2") | Search modes - Boolean/Phrase | 156,272 |
| S5 | S1 OR S2 OR S3 OR S4 | Search modes - Boolean/Phrase | 208,326 |
| S4 | TI ( ((elevat* or increase* or high or rais* or rising) N2 (bp or dbp or hbp or sbp)) ) OR AB ( ((elevat* or increase* or high or rais* or rising) N2 (bp or dbp or hbp or sbp)) ) | Search modes - Boolean/Phrase | 4,610 |
| S3 | TI ( ((blood or arterial or diastolic or systolic) N3 (pressure)) ) OR AB ( ((blood or arterial or diastolic or systolic) N3 (pressure)) ) | Search modes - Boolean/Phrase | 90,124 |
| S2 | TI ( hypertens* or prehypertens* or high blood pressure or HTN or HBP ) OR AB ( hypertens* or prehypertens* or high blood pressure or HTN or HBP ) | Search modes - Boolean/Phrase | 109,350 |
| S1 | (MH "Blood Pressure+") OR (MH "Hypertension+") | Search modes - Boolean/Phrase | 125,731 |

Cochrane Library

April 25, 2022

#1 exp hypertension OR exp blood NEAR pressure 2090

#2 hypertens* OR prehypertens* OR high NEAR blood NEAR pressure OR htn OR hbp 85476

#3 (blood OR arterial OR diastolic OR systolic) AND pressure 122176

#4 ((elevat$ or increas$ or high or rais$ or rising) (bp or dbp or hbp or sbp)) 7040

#5 #1 OR #2 OR #3 OR #4 162451

#6 diabetes NEAR mellitus OR diabetes NEAR type1 OR diabetes NEAR mellitus NEAR type2 OR exp diabetes NEAR mellitus NEAR type1 OR exp diabetes NEAR mellitus NEAR type2 71474

#7 diabet* OR prediabet* OR "blood sugar" OR "diabetes mellitus" OR "type 1 diabetes" OR "type 2 diabetes" OR "type 1 diabetes mellitus" OR "type 2 diabetes mellitus" OR t1d OR t2d OR t1dm OR t2dm OR "non insulin* depend*' OR "noninsulin* depend*' OR "noninsulindepend*" OR "non insulin?depend*" OR niddm OR "insulin* depend*" OR "insulindepend*" OR iddm 111676

#8 #6 OR #7 111676

#9 #5 OR #8 245410

#10 exp telemedicine OR distance NEAR counseling OR remote NEAR consultation 621

#11 telemed* OR teleadminist* OR teleadvice OR teleassess* OR telecare OR telechat* OR teleconf* OR teleconsult* OR telecounsel* OR teledeliv* OR telehealth* OR teleinterv* OR telemanag* OR telemonit* OR telenurs* OR telepharm* OR televisit* OR teletherap* OR videochat* OR videotelephon* 9213

#12 (econsult* OR "e consult*" OR ecounsel* OR "e counsel*" OR ehealth* OR "e health*" OR einterv* OR "e interv*" OR etherap* OR "e therap*" OR mhealth* OR "m health*" OR mobile) AND health* 15296

#13 (comput* OR distance OR internet OR phone OR online OR remote OR tele* OR video OR virtual OR web) NEAR/2 (administ* OR advice OR assess* OR care OR chat* OR confer* OR consult* OR counsel* OR deliver* OR health* OR interv* OR manag* OR medic* OR monit* OR nurs* OR pharm* OR therap* OR visit*) 44142

#14 app OR apps OR facetime* OR helpline* OR "store and forward*" OR "store forward*" OR skype* OR video* OR zoom OR "webbased tool" OR "web-based tool*" OR "voice over" OR voiceover OR voip 35731

#15 #10 OR #11 OR #12 OR #13 OR #14 81725

#16 Health NEAR plan NEAR implementation OR program NEAR evaluation 11106

#17 intervention* OR program* OR develop* OR implement* OR campaign OR evaluat* OR assess* OR pilot OR impact 1221845

#18 #16 OR #17 1221845

#19 #9 AND #15 AND #18 11633

#20 limit #19 to (english AND articles) with Cochrane Library publication date Between Jan 2017 and Apr 2022 396

Web of Science

April 25, 2022

1 TS=(hypertension OR blood pressure) 809,714

2 TS=(hypertens* or prehypertens* or "high blood pressure" or HTN or HBP) 604,048

3 (TI=(hypertens* or prehypertens* or "high blood pressure" or HTN or HBP)) OR AB=(hypertens* or prehypertens* or "high blood pressure" or HTN or HBP) 501,376

4 TS=((elevat$ or increas$ or high or rais$ or rising) NEAR/2 (bp or dbp or hbp or sbp)) 16,065

5 (TI=(blood or arterial or diastolic or systolic NEAR/3 pressure)) OR AB=(blood or arterial or diastolic or systolic NEAR/3 pressure) 2,176,307

6 #1 OR #2 OR #3 OR #4 878,246

7 TS=(diabetes mellitus/ or diabetes mellitus, type 1/ or diabetes mellitus, type 2/ or exp Diabetes Mellitus, Type 1/ or exp Diabetes Mellitus, Type 2/) 324,324

8 (TI=(diabet* or prediabet* or "blood sugar" or "diabetes mellitus" or "type 1 diabetes" or "type 2 diabetes" or "type 1 diabetes mellitus" or "type 2 diabetes mellitus" or T1D or T2D or T1DM or T2DM or "non insulin* depend*" or "noninsulin* depend*" or "noninsulindepend*" or "non insulin?depend*" or NIDDM or "insulin* depend*" or "insulindepend*" or "insulin?depend*" or IDDM)) OR AB=(diabet* or prediabet* or "blood sugar" or "diabetes mellitus" or "type 1 diabetes" or "type 2 diabetes" or "type 1 diabetes mellitus" or "type 2 diabetes mellitus" or T1D or T2D or T1DM or T2DM or "non insulin* depend*" or "noninsulin* depend*" or "noninsulindepend*" or "non insulin?depend*" or NIDDM or "insulin* depend*" or "insulindepend*" or "insulin?depend*" or IDDM) 829,023

9 #7 OR #8 864,625

10 #6 OR #9 1605,933

11 TS=(Telemedicine/ or Distance Counseling/ or Remote Consultation/) 32,981

12 (TI=(telemed* or teleadminist* or teleadvice or teleassess* or telecare or telechat* or teleconf* or teleconsult* or telecounsel* or teledeliv* or telehealth* or teleinterv* or telemanag* or telemonit* or telenurs* or telepharm* or televisit* or teletherap* or videochat* or videotelephon*)) OR AB=(telemed* or teleadminist* or teleadvice or teleassess* or telecare or telechat* or teleconf* or teleconsult* or telecounsel* or teledeliv* or telehealth* or teleinterv* or telemanag* or telemonit* or telenurs* or telepharm* or televisit* or teletherap* or videochat* or videotelephon*) 36,056

13 (TI=(eConsult* or e-consult* or ecounsel* or e-counsel* or eHealth* or e-Health* or einterv* or e-interv* or etherap* or e-therap* or mHealth* or m-Health* or mobile health*)) OR AB=(eConsult* or e-consult* or ecounsel* or e-counsel* or eHealth* or e-Health* or einterv* or e-interv* or etherap* or e-therap* or mHealth* or m-Health* or mobile health*) 49,855

14 (TI=((comput* or distance or internet or phone or online or remote or tele* or video or virtual or web) NEAR/2 (administ* or advice or assess* or care or chat* or confer* or consult* or counsel* or deliver* or health* or interv* or manag* or medic* or monit* or nurs* or pharm* or therap* or visit*))) OR AB=((comput* or distance or internet or phone or online or remote or tele* or video or virtual or web) NEAR/2 (administ* or advice or assess* or care or chat* or confer* or consult* or counsel* or deliver* or health* or interv* or manag* or medic* or monit* or nurs* or pharm* or therap* or visit*)) 300,617

15 (TI=(App or apps or facetime* or helpline* or store-and-forward* or store-forward* or skype* or video* or zoom or "webbased tool" or "web-based tool*" or voice-over or voiceover or VoIP)) OR AB=(App or apps or facetime* or helpline* or store-and-forward* or store-forward* or skype* or video* or zoom or "webbased tool" or "web-based tool*" or voice-over or voiceover or VoIP) 497,461

16 #11 OR #12 OR #13 OR #14 OR #15 813,202

17 TS=(Health plan implementation/ or program evaluation/) 219,672

18 (TI=(intervention* or program* or develop* or implement* or campaign or evaluat* or assess* or pilot or impact)) OR AB=(intervention* or program* or develop* or implement* or campaign or evaluat* or assess* or pilot or impact) 22,074,972

19 #17 OR #18 22,075,851

20 #10 AND #16 AND #19 15,127

21 Refine by PY, DT, Language: #44 AND #34 AND #16 and 2022 or 2021 or 2020 or 2019 or 2018 or 2017 (Publication Years) and Articles (Document Types) and English (Languages) 5322

Google Scholar

April 25, 2022

1. Time range 2017 to current
2. hypertension; blood pressure 437,000
3. ~hypertension or ~prehypertension or high blood pressure or HTN or HBP 719
4. ~elevate or ~increase or high or ~raise or ~rising bp or dbp or hbp or sbp 3060
5. blood or arterial or diastolic or systolic or pressure 17,200
6. diabetes mellitus/ or diabetes mellitus, type 1/ or diabetes mellitus, type 2/ or exp Diabetes Mellitus, Type 1/ or exp Diabetes Mellitus, Type 2 16,700
7. implementing telemedicine interventions for hypertension or diabetes management 17,500
8. Extract first 5 pages 50
9. Manually exclude review articles 20

This is a Multimedia Appendix to a full manuscript published in the J Med Internet Res. For full copyright and citation information see https://dx.doi.org/10.2196/jmir.42134
